# Nurses’ eating habits in Lebanon during the economic and health crises: a cross-sectional study

**DOI:** 10.1186/s13690-021-00775-1

**Published:** 2022-01-14

**Authors:** Rita Georges Nohra, Elissa Naim, Taghrid Chaaban, Monique Rothan-Tondeur

**Affiliations:** 1 Nursing Sciences Research Chair, Laboratory Educations and Health Practices (LEPS), (EA 3412), UFR SMBH, F-93017, Université Sorbonne Paris Nord, Bobigny, France; 2 Hôtel-Dieu de France Hospital, Beirut, Lebanon; 3 Faculty of Public Health, Lebanese University, Branch II, Fanar, Lebanon; 4 Doctoral School of Sciences and Technology (EDST), Lebanese University, Hadath, Lebanon; 5 INSPECT-LB: Institut National de Santé Publique, Epidémiologie Clinique et Toxicologie, Beirut, Lebanon; 6 Faculty of Nursing and Health Sciences , Islamic University of Lebanon, Beirut, Lebanon; 7 APHP, Nursing Sciences Research Chair, Paris, France

**Keywords:** COVID-19, Eating habits, Economic crisis, Frontline nurses, Lebanon, Stress

## Abstract

**Background:**

Nurses face multiple stressors that can influence their lifestyle, thus affecting their health status. Scarce are the scientific data on the nutritional status of nurses, especially during health crises. The aim of this study was to assess the impact of the COVID-19 pandemic on the eating habits of hospital nurses in the context of an exceptional economic situation in Lebanon.

**Methods:**

A cross-sectional study was conducted using a web-based questionnaire, targeting a non-random sampling of frontline nurses using the snowball technique. Descriptive and bivariate analyses were carried out. The population of the study included all registered nurses working in the Lebanese hospitals. A total of 533 nurses completed the questionnaire; 500 surveys were selected after excluding the ones presenting conditions that may affect their eating behavior.

**Results:**

The majority of the respondents were women (78.6%) with a mean age of 33 years [18-60] [SD,7.44 years]. Most of them (57.6%) had a crowding index ≥1. The consumption of different food groups decreased during these crises. There was a significant correlation between stress and deterioration of healthy food consumption, which provides beneficial nutrients and minimizes potentially harmful elements, especially for meat (OR 2.388, CI 1.463 to 3.898, *P *< 0.001). The decrease in monthly income showed a real impact on the consumption of healthy food such as meat (OR 2.181, CI 1.504 to 3.161, *P *< 001), fruits (OR 1.930, CI 1.289 to 2.888, *P *= 0.001), and milk and dairy products (OR 1.544, CI 1.039 to 2.295, *P *= 0.031).

**Conclusions:**

The pandemic and in particular the economic crisis has changed the consumption of healthy food among hospital nurses in Lebanon. Similar research and support may be extended to include other frontline health care workers.

## Background

The pandemic of COVID-19 is a major global public health emergency. As of August 2020, there have been 17,918,582 confirmed cases worldwide, with 691,013 deaths. Regarding the Lebanese situation, 13,687 confirmed cases and 138 deaths have been reported [8]. The rapidly evolving pandemic has stressed the entire health care system worldwide, especially in Lebanon where an extensive economic crisis has been striking the country since February 2020.

An overwhelmed healthcare system can severely compromise the well-being of its frontline workers [15]. Nurses constitute the largest part of the healthcare workforce in an epidemic [28], and they undertake most of the tasks related to infectious disease containment [35]. The intensive work during epidemics drains health care providers physically and emotionally [20]. High workloads, working hours, low staffing, stress, and economic barriers can influence nurses’ health by changing their lifestyle behaviors [6], and nutrition is well recognized as a central component of a healthy lifestyle [33].

Nutritious and healthy food is the one that provide beneficial nutrients (e.g. vitamins, minerals, essential amino acids, essential fatty acids, dietary fiber) and minimizes potentially harmful elements (e.g. anti-nutrients, quantities of sodium, saturated fats, sugars) (Neufeld et al., [37]). The relation between healthy food and immunity has been largely discussed in the literature [21], and a particular insight was addressed lately for their implications with COVID-19. Adequate nutrition is required for the immune system cells to function, with a higher demand for energy during infection. Specific nutrients (vitamins A, B6, B12, C and D plus copper, folate, iron, selenium, and zinc) are also needed to fulfill particular roles as for the production and stimulation of immune cells (Lockyer, [38]). Consequently, deficiencies in these micronutrients can also have effects on reducing the immunity response (Gombart et al., [39]). Perceived stress has a direct impact on eating behavior (Barrington et al.,[40]). If stress levels remain high, a person often feels hungry and eats more, especially more of the foods that are high in sugar and fat. This pattern leads to a greater likelihood of fat deposition to cause obesity. The final part of this cycle is that obesity is linked to the development of other chronic conditions such as cardiovascular disease, Type 2 diabetes, and osteoarthritis (CDC, [41]). Malnutrition has a considerable impact on the health and productivity of an individual, which affects the whole country’s development [14]. In 2017, 11 million deaths worldwide were attributable to dietary factors [2]. Hence, the prevalence of poor dietary intake has increased throughout the 21st century [12] placing a poor diet ahead of any other risk factor for death in the world [2]. The Mediterranean diet, a dietary pattern followed in Lebanon, is characterized by the consumption of cereals preferably as whole grains, legumes, nuts, vegetables, and fruits, in high amount and frequency. Several studies have shown that the consumption of foods included in this typical diet has anti-inflammatory and immune-modulating properties (Barrea et al., [42]). Moreover, the Mediterranean diet has shown to reduce stress hormone response to acute stress (Carvalho et al., [43]; Shively et al., [44]).

Scarce are the scientific data done on the nutritional status of nurses, especially during health crises. Most published research on hospital nurses focuses on patient safety, workload, and nurse performance [16, 24, 25], with relatively few studies on lifestyle and preventive health behaviors, which makes it difficult to define appropriate intervention strategies for them. Facing the current situation in Lebanon, most nurses have emigrated, and others have resigned. As for the rest, there is an absence of epidemiological data on their health status including their eating habits. Therefore, the aim of our study is to assess the impact of the COVID-19 pandemic, in the context of an exceptional economic situation in Lebanon, on the eating habits of Lebanese hospital nurses.

The analysis of food consumption behavior is particularly complex. From the beginning of studies, the dominant approach has been behaviorist, considering that environmental stimuli lead to behavioral responses. Very quickly, the limits of the behaviorist approach, and in particular its insufficient consideration of the consumer’s psychology, led specialists to focus more on individual factors [19]. According to the POS paradigm, consumer perceptions, evaluations, and behaviors are the results of three types of factors: the characteristics of the object, the characteristics of the person, and the characteristics of the environment [18]. Over the years, several models have been developed based on this paradigm, such as the “Food Perception Model” which distinguishes situational from other environmental factors [32]. According to this model, the situation should be considered not only as a direct determinant of consumption, but also because of its interactions with individual factors [32]. Given the specificities of the hospital environment, we have chosen the “Food Perception Model” basis to highlight the distinction between situation and environment factors.

## Methods

### Study design and participants

This cross-sectional study was conducted from June the 1st to August the 4th of the year 2020. The date of August 4, 2020 has marked the history of Lebanon with the dramatic Beirut port blast. Thus, to avoid any possible factors that might affect the participants’ answers, the survey was ended right after it.

**Fig. 1:**
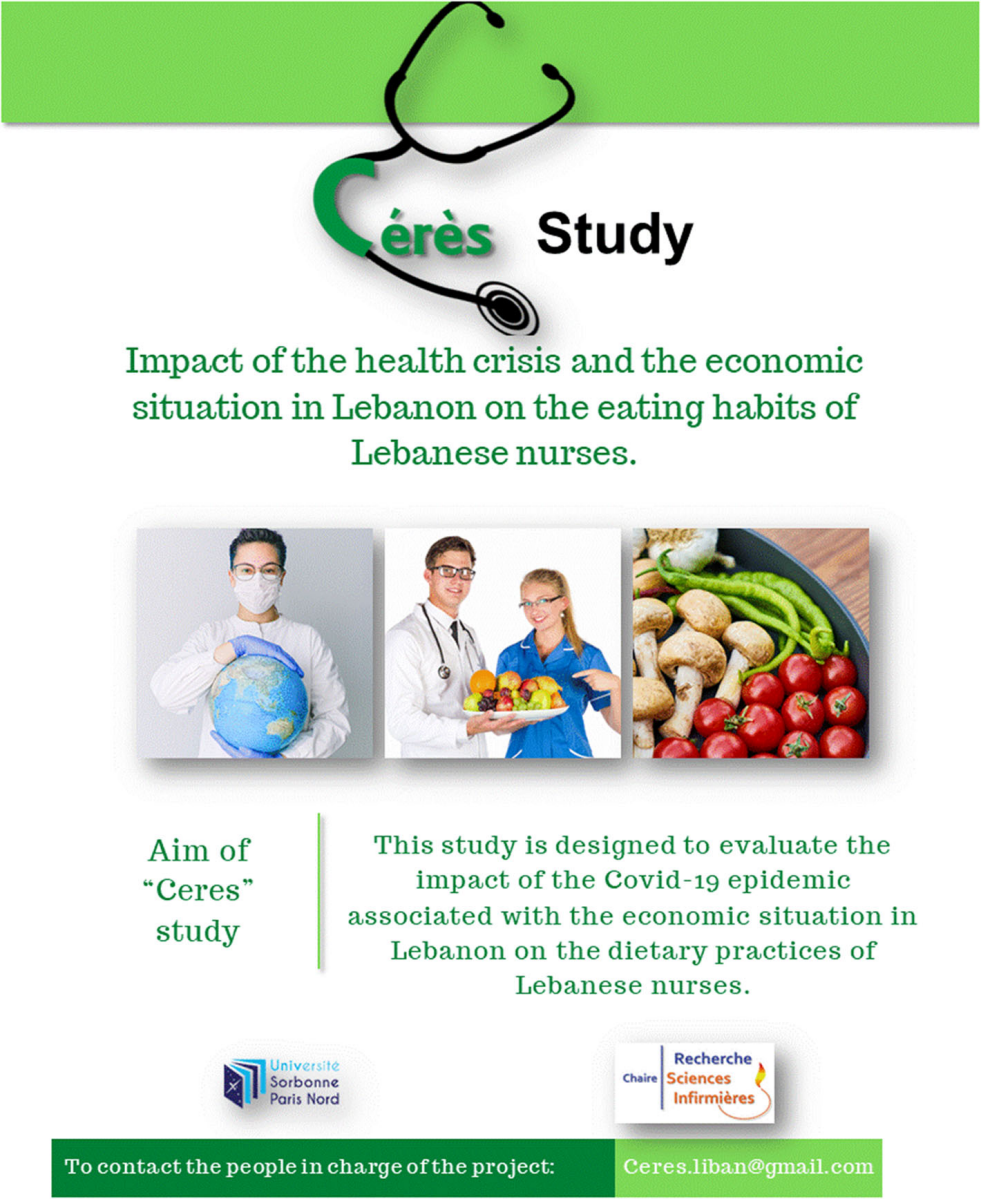
Flyer for social media

All registered nurses, affiliated to the Lebanese Order of Nurses, working in the Lebanese hospitals and of all ages, were invited to participate in this study, except for the nurses who were pregnant, on maternity or sick leave, and not members of the national Order. Moreover, nurses with chronic diseases that could influence their diet were also excluded from the final analysis. A non-random sampling was conducted. Nurses were selected using the snowball technique in three successive stages. In the first wave, the distribution of an online questionnaire was executed by the Lebanese Order of Nurses to all nurses affiliated to the order and working in the Lebanese hospitals. The snowball method was also used via WhatsApp. In the second wave, a flyer was posted on social networks (Facebook and LinkedIn) to motivate nurses to fill out a questionnaire (Fig. 1). In the third wave, the nursing directors of all Lebanese hospitals were contacted and asked to distribute the questionnaire to their fellows through the WhatsApp group of each hospital.

### Procedures and outcomes

A questionnaire was distributed via the web through a link in three languages: French, English, and Arabic. The questionnaire collected information on socio-demographic and economic characteristics, nurses’ eating habits before and during the health and economic crises in Lebanon, and the level of coping through Brief COPE. It is worth noting that this questionnaire was disseminated three months after the start of both crises which were relatively simultaneous; participants were asked to compare their lifestyle habits during these two periods (before and during the crises). The questionnaire was pre-tested with 18 nurses.

Economic characteristics were evaluated based on the monthly salary, decrease in salary according to the economic crisis, and the number of people under the nurses’ financial responsibility. The crowding index (number of people living in the same house/number of rooms in the house) was also calculated to define the socio-economic level. An index > 1 indicates an overcrowded house with few economic resources [13].

Dietary intakes before and during both crises were measured through a validated questionnaire from Samhat et al. [29] including seven food groups: cereals and starches, fruits, vegetables, milk and dairy products, meat, fat, sweets and four types of beverages: caffeinated drinks, soft drinks, juices, and energy drinks. The Likert scale was employed to measure the participants’ daily food and beverage intake were: never or rarely, once, two to three times, four to five times, six times a day or more. According to the results, participants were classified into two groups as presenting a deterioration in healthy food consumption or not. Deterioration was detected when the consumption of healthy food groups was less frequent during both crises compared to the period before.

Furthermore, information regarding smoking, alcohol intake, water intake, taking a meal break, eating main meals and snacks, ordering delivery during working hours, doing physical activity, and body mass index (BMI) were collected using the same validated questionnaire [29].

Brief COPE encompasses 28 multidimensional items in 14 subscales, which measures the strategies used in response to stressors. There are 8 items that measure coping strategies presumed to be adaptive and 6 items that focus on coping strategies presumed to be maladaptive or avoidance [5]. Summary scores were generated by adding the subscale scores in each domain. A higher summary score indicates a higher level of adaptation or avoidance.

Two semi-open-ended questions were added to explore the participants’ activities during the confinement period and to collect suggestions reported by the participants.

The data was automatically saved on the web, downloaded in Excel format, and analyzed using SPSS 21 software. All results were cross-checked to detect errors or inconsistencies.

### Statistical analysis

A descriptive analysis was performed to summarize the data on nurses’ demographics variables and to present the economic level, eating habits before and during the two crises, and the used coping strategies.

Bivariate analysis was carried out to assess the significant differences using student’s t-test for continuous variables and χ2 test for categorical variables. In order to determine the factors influencing the deterioration of dietary intakes, an analysis was conducted by comparing food groups’ consumption with socio-economic variables. Body Mass Index (BMI) was calculated. Finally, the impact of adaptation level to stress on eating habits during the two crises was determined. Statistical significance was set at *P *< 0.05. Bivariate analysis was used to estimate odds ratios (ORs) and corresponding 95% confidence intervals (CIs). All statistical analyses were done using SPSS 21.

## Results

A total of 533 nurses completed the questionnaire of which 500 (representing 3% of the Lebanese nursing population) were selected after excluding the ones suffering from chronic diseases or allergies which may affect their eating behavior. The majority of nurses were women (78.6%, *n *= 393), married (55.6%, *n *= 278), the mean age was 33 years [18-60] [SD 7.44 years] with 11 years [1-38] [SD 7 years] of experience. Most of the respondents had a university degree (78%, *n *= 390) and 23% held a master’s degree (*n *= 115). The majority were bedside nurses (74.8%, *n *= 374), responsible of 10 patients [1-75] [SD 9 patients], and 43.4% were working day and night (*n *= 217). Among the participants, 53.2% took care of COVID patients (*n *= 266), while only 13.4% were working in a special COVID-19 unit (*n *= 67). The monthly income of 88.4% of the respondents was less than 1,600$ (1$=1,500LL) (*n *= 442) and 58.6% reported a decrease in their salary due to the economic crisis (*n *= 293). Moreover, results have shown that 57.6% of the nurses had a crowding index ≥1 (*n *= 288), with 45% (*n *= 225) of the nurses having three or more people in their household under their financial responsibilities. While 40.6% (*n *= 203) of the participants stated that they were satisfied with their work, 80.6% (*n *= 403) reported being stressed during this period, among which 96.5% (*n *= 389) attributed this stress to the economic crisis and 75.9% (*n *= 306) attributed it to the health crisis. In addition, 70.6% (*n *= 353) of the nurses expressed a change in their eating habits, for which the participants blamed the economic crisis (79.9%, *n *= 282). Indeed, 76.2% of the nurses (*n *= 381) declared having financial difficulties in shopping for healthy food. Hence, among the 68.6% (*n *= 242) who reported an unfavorable change, half of them declared taking corrective measures mainly through self-management of their nutrition plan (57.4%, *n *= 139) and physical activities (37.1%, *n *= 90). However, an increase of 67% (*n *= 335) of sedentary was seen among the participants during this period (Table 1).

**Table 1:** Sociodemographic and other characteristics of Lebanese hospital nurses (*N *= 500)

Characteristic	Incidence
Gender, n(%)	
Male	107 (21.4)
Female	393 (78.6)
Age, mean (SD)	33 (7.44)
Marital status, n(%)	
Single	210 (42)
Married	278 (55.6)
Divorced	12 (2.4)
Diploma, n(%)	
Higher technical	110 (22)
Bachelor’s degree	275 (55)
Master’s degree	115 (23)
Clinical experience (years), mean (SD)	11 (7)
Professional title, n(%)	
Registered Nurse	374 (74.8)
Head of Department	77 (15.4)
Supervisor	45 (9)
Working shifts, n(%)	
Always day	212 (42.4)
Always night	71 (14.2)
Day and night	217 (43.4)
Number of patients/shift, mean (SD)	10 (9)
Working units, n(%)	
COVID-19 special units	67 (13.4)
Other units	433 (86.6)
Caring for COVID-19 patients, n(%)	
Yes	266 (53.2)
No	234 (46.8)
Overall satisfaction at work, n(%)	
Not satisfied	118 (23.6)
Somewhat satisfied	179 (35.8)
Satisfied	173 (34.6)
Very satisfied	30 (6)
Feeling stressed, n(%)	
Yes	403 (80.6)
No	97 (19.4)
Stress triggers, n(%)	
Economic crisis	389 (96.5)
COVID-19 contamination risk	306 (75.9)
Wearing PPE (Personal Protective Equipment)	204 (50.6)
Stigmatizing attitudes towards nurses	220 (54.6)
Other	291 (72.2)
Monthly income (LBP) (1,500 LBP =1$), n(%)	
< 1 500 000 LL	257 (51.4)
1 500 000 LL - 2 400 000 LL	185 (37)
2 500 000 LL -3 400 000 LL	41 (8.2)
>3 500 000 LL	17 (3.4)
Decrease in income due to the economic crisis, n (%)	
Yes	293 (58.6)
No	207 (41.4)
Crowding Index, n(%)	
≥ 1	288 (57.6)
< 1	212 (42.4)
People under nurse financial charge, n(%)	
< 3	275 (55)
≥ 3	225 (45)
Financial difficulties to buy healthy Food, n(%)	
Yes	381 (76.2)
No	119 (23.8)
Changing in weight, n(%)	
Losing	169 (33.8)
Same	170 (34)
Gaining	161 (32.2)
Changing in eating habits, n(%)	
Yes	353 (70.6)
No	147 (29.4)
Nature of the change in eating habits, n(%)	
Good change	111 (31.4)
Unfavorable	242 (68.6)
Corrective actions (for the unfavorable change), n(%)	
None	111 (45.8)
Workout	90 (37.1)
Dietary counseling	45 (18.5)
Self-management	139 (57.4)
Reasons of changing in eating habits, n(%)	
Economic crisis	282 (79.9)
Lockdown	232 (65.7)
Wearing PPE	120 (33.9)
Boosting immune system	152 (43.05)
Other (workload, …)	252 (71.3)

Participants were asked if they see themselves as fat, thin, or as having normal weight. Hence, the participants’ perception of their body image was reported as normal for 68.8% (*n *= 344) before confinement, which decreased to 61.8% (*n *= 309) during the crises. According to the international classification of the BMI, 3.8% (*n *= 19) were classified as underweight (BMI <18.5) before the confinement which increased to 4.6% (*n *= 23) during the crises period, and no change was seen in the other three classifications. However, 66% (*n *= 330) of the nurses reported a change in their weight during the crises among which 51.2% (*n *= 169) reported a weight loss (3.8 Kg [-2-20 kg] [SD 3.14 Kg]) while 48.7% (*n *= 161) reported a weight gain (4.31 Kg [0.5-16 kg] [SD 3 Kg]). Furthermore, the number of participants consuming three main meals per day decreased from 18.6% (*n *= 93) to 13.6% (*n *= 68) during the crises. Respondents (14.4%, *n *= 72) who reported not to take their meal breaks at work increased to 20.8% (*n *= 104) during the crises period. Nevertheless, participants who frequently used to order deliveries decreased by half (22.8%, *n *= 114 and 10.6%, *n *= 53 respectively). Moreover, heavy smokers increased from 2.8% (*n *= 14) to 5.4% (*n *= 27) during the crises. Nonetheless, there was no change in daily water consumption, although almost 64.8% (*n *= 324) of this population does not drink enough (Table 2).

**Table 2:** Lifestyle perception and eating habits among Lebanese hospital nurses (*N *= 500)

Variables	Before crises	During crises
Weight perception, n(%)		
I am good	344 (68.8)	309 (61.8)
I am overweight	122 (24.4)	136 (27.2)
I am underweight	34 (6.8)	55 (11)
BMI, mean (SD)	25.76 (5.1)	25.64 (4.933)
Weight (Kg), mean (SD)	70.23 (16)	69.94 (15.82)
Physical activities, n(%)		
Inactive (no physical activity outside the profession)	161 (32.2)	269 (53.8)
Moderately active (at least twice a week)	269 (53.8)	193 (38.6)
Very active (more than three times a week)	70 (14)	38 (7.6)
Smoking, n(%)		
Non-smoker or ex-smoker	339 (67.8)	332 (66.4)
Light or moderate smoker (<1pack.day)	147 (29.4)	141 (28.2)
Heavy smoker (> 1 pack/day)	14 (2.8)	27 (5.4)
Alcohol consumption, n(%)		
Never	461 (92.2)	460 (92)
Once a day (≤ 7 times a week for women) twice a day (≤14 times a week for men)	35 (7)	35 (7)
> once a day (> 7 times a week for women) > twice a day (>14 times a week for men)	4 (0.8)	5 (1)
Fluid intake ≥2 L of water per day, n(%)		
Never	76 (15.2)	74 (14.8)
Sometimes	258 (51,6)	250 (50)
Frequently	104 (20.8)	113 (22.6)
Every day	62 (12.4)	63 (12.6)
Consumption of the 3 main meals (Breakfast, lunch, dinner) per day, n (%)		
Never	40 (8)	67 (13.4)
Sometimes	232 (46.4)	257 (51.4)
Frequently	135 (27)	108 (21.6)
Every day	93 (18.6)	68 (13.6)
Taking meal breaks during duty, n(%)		
Never	72 (14.4)	104 (20.8)
Sometimes	268 (53.6)	275 (55)
Frequently	107 (21.4)	81 (16.2)
Every day	53 (10.6)	40 (8)
Snacks consumption in between meals, n(%)		
Never	96 (19.2)	155 (31)
Sometimes	295 (59)	262 (52.4)
Frequently	89 (17.8)	67 (13.4)
Every day	20 (4)	16 (3.2)
Order manakesh, fries, chips, falafels, shawarma, burgers, pizzas, desserts during duty, n(%)		
Never	70 (14)	221 (44.2)
Sometimes	301 (60.2)	222 (44.4)
Frequently	114 (22.8)	53 (10.6)
Every day	15 (3)	4 (0.8)

**Fig. 2:**
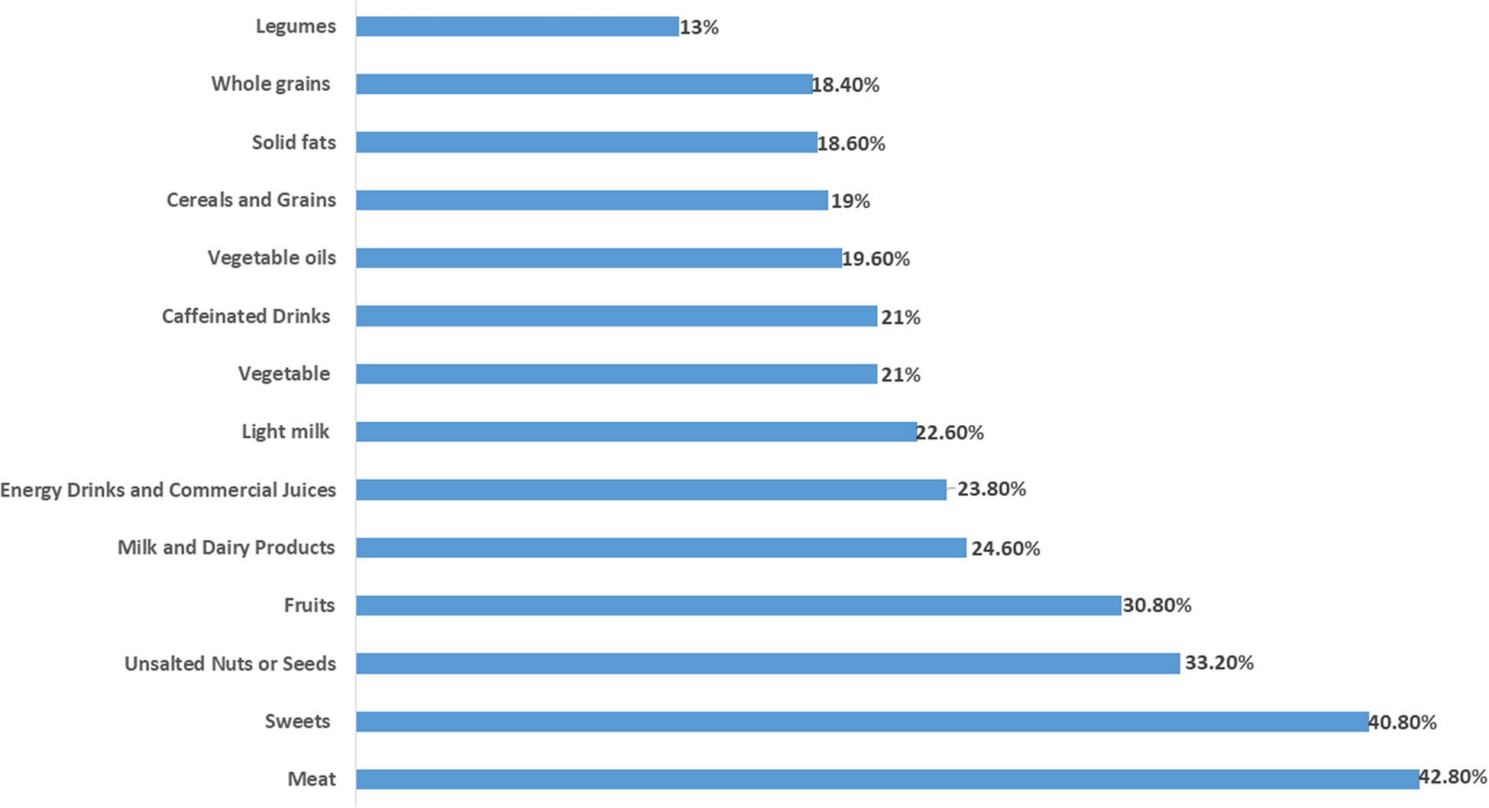
The decrease in the consumption of different food groups (%)

Consumption of the different food groups decreased during these crises. Some decreases indicate a deterioration in nurses’ healthy eating habits such as meat (42.8%, *n *= 214), fruits (30.8%, *n *= 154), milk and dairy products (24.6%, *n *= 123), and vegetables (21%, *n *= 105). Nevertheless, other decreases favor an improvement in their eating habits such as a decrease in the consumption of sweets (40.8%, *n *= 204) and soft drinks (23.8%, *n *= 119) (Fig. 2). No significant relationship was found between the deterioration of healthy eating habits, age, and years of experience. However, nurses who had worked with patients with COVID-19 consumed significantly less fruits (OR 1.559, CI 1.059 to 2.295, *P *= 0.022) and vegetables (OR 1.65, CI 1.06 to 2.568, *P *= 0.0262). In addition, results showed that there was a significant relationship between stress and the deterioration of healthy eating habits, especially for meat (OR 2.388, 95% CI, 1.463 to 3.898, *P *< 0.001), fruits (OR 1.763, CI 1.042 to 2.983, *P *= 0.032), and vegetables (OR 1.911, CI 1.019 to 3.585, *P *= 0.041). Similarly, it was found that the decrease in monthly income has a significant impact on the consumption of meat (OR 2.181, CI 1.504 to 3.161, *P *< 000), fruits (OR 1.930, CI 1.289 to 2.888, *P *= 0.001), milk, and dairy products (OR 1.544, CI 1.039 to 2.295, *P *= 0.031) (Table 3).

**Table 3:** Association between socioeconomic, demographic and stress with eating habits: Bivariate analysis

Variables		Decrease consumption							
		Meat		Milk and dairy products		Fruits		Vegetables	
		Incidence, n	*P* value	Incidence, n	*P* value	Incidence, n	*P* value	Incidence, n	*P* value
Gender	Male	49	0.480	36	0.410	36	0.406	32	**0.011**
	Female	165		116		117		73	
Age	< 26	63	0.158	41	0.335	42	0.577	25	0.347
	26 - 32	56		37		38		28	
	32 -39	51		42		39		26	
	>39	44		32		34		26	
Clinical experience (years)	≤10	116	0.817	81	0.654	82	0.739	61	0.445
	>10	98		71		71		44	
Caring of COVID- 19 patients	Yes	91	0.097	63	0.113	60	**0.022**	39	**0.026**
	No	123		89		93		65	
Stress	Yes	188	**0.001**	22	0.066	132	**0.032**	92	**0.041**
	No	26		22		21		13	
Monthly income	≤2500	200	**0.002**	137	0.424	136	0.812	91	0.533
	>2500	14		15		17		14	
Decrease in income	Yes	148	**0.001**	100	**0.031**	106	**0.001**	64	0.582
	No	66		52		47		41	

Lastly, 71 participants shared their suggestions in response to their situation where the majority expressed the urge of reconsidering their salaries and the workload. Considering the hard work they are doing, the participants asked to increase, or even just to receive their salary on time, which in their opinion has lost its value in this time of crisis. Similarly, most participants requested hospitals to provide them with healthy meals free of charge or at affordable prices.

## Discussion

This study was based on the four elements of the “Food Perception Model” [32]: the individual, the environment, the context, and the food that can affect food consumption.

Under the category of the “Individual” element, the demographic and the psychological variables were distinguished. Studies have shown that gender and age influence eating behaviors and cravings [1]. However, no significant difference was identified between the variation in consumption of healthy food, sex, and age of participants. Nevertheless, the results of this study showed a significant correlation between the deterioration of healthy food intake and the stress perceived by the participants. This can be justified by the mechanism in which stress influences food choices involving hormonal interactions and metabolic processes, as well as individual differences in the psychological and neurochemical response to stress and diet [34].

In the frame of the “Environmental” element, the economic and health crises which have directly impacted nurses’ food consumption were determined. The economic status of the nurses was correlated with a deterioration in food consumption. Nurses reported a decrease in their monthly income, which had a significant impact on their food intake. Participants declared having financial difficulties that impeded them from buying healthy food especially following the uncontrolled increase in the food price in Lebanon. Note that the value of the Lebanese Pound (LBP) shows a daily major fluctuation with three different values in the market. Hence, the official original Lebanese currency of 1,507 LBP for $ 1 is faced by a value of 3,900 LBP in the banking sector and an hourly fluctuating value of 7,800 LBP for 1 $ (November 17, 2020) in the black market. Consequently, a beginner nurse earns $ 200 per month. Indeed, studies showed that participants from low-income countries were more likely to consume low-quality food [3]. Moreover, the health crisis has already taken its part in the food consumption of the Lebanese nurses. Henceforth, a significant relationship was seen between nurses working with COVID patients and the deterioration in their intake of fruits and vegetables. It is an alarming situation since a decrease in the consumption of these antioxidant-rich food groups can affect their immunity, thus threatening their health [4]. In addition, both economic and health crises had a negative effect on nurses’ physical activity and weight. Thus, alarming results were observed among nurses who are supposed to be the role model in their profession and in front of the patients, increasing the risk of chronic disease prevalence which is dangerous in times of health crisis like COVID-19 [30].

In terms of the “Context” element of the Food Perception Model, the healthcare system has a constantly challenging context (place, shifts, and time) which has a direct impact on the medical staff’s eating habits [22]. The kitchen of the unit floor is the place where the nurses take their lunch, so they are predisposed to cut their meals several times to meet patient requests, thus affecting their eating habits. Indeed, some of our participants raised the flag for an urgent need of a calm and dignified place where they can have their meals. As for the nurse-to-patient ratio, no-load limit was set in Lebanon, due to staffing shortages affecting hospitals. Rassin & Silner [27] found that an increase in the nurse-to-patient ratio from 1:4 to 1:6 raised the patient mortality rate by 7%, and with further increase in nurse-patient ratio to 1:8, the mortality rate increased to 14%. Nurses in this study declared working with up to 10 patients per shift. This can seriously compromise not only patients’ safety [31] but also nurses’ satisfaction and emotional health [17]. Moreover, the working hours have a direct effect on the nurses’ lifestyle. The majority of participants’ work day and night. Hence, recent studies have shown that shift work presents a major source of stress which affects the circadian rhythm, thus the regularity, the quality, and the number of meals taken during different shifts [36]. Certainly, this may explain the decrease in dietary fiber consumption (vegetables and fruits), limited meals, and food choices among our participants.

As for the “Food” element, in a country where almost everything is imported, the price of food products has increased by 72% between October 2019 and the end of May 2020 [10]. For example, the price of beef meat increased by 111% compared to the same period in 2019 [7], which may explain the decrease in meat intake reported by our participants. Moreover, Lebanon is the second country in the world with the highest price of a liter of milk after Taiwan, being 3.07$ (as of November 18, 2020) [26]. The national Consultation and Research Institute (CRI) indicates that in May 2020, the price of powdered milk increased by 69% compared to May 2019, and that of cheese increased by 98% [7]. Hence, participants’ milk consumption reduced compared to before the crisis. In addition, the decrease in the consumption of vegetables and fruits can be explained in particular by the price increase of more than 200% of this food category [11]. Moreover, a decrease in the consumption of all food groups was indicated in this population, mostly in the categories of meat, milk and dairy products, fruits, vegetables, whole grains, and vegetable oils. The results of a recently published study showed that subjects exposed to the coronavirus with no clinical symptoms of COVID-19 consumed significantly more dairy products than the ones with clinical symptoms of COVID-19 [23]. This might be due to the presence of probiotics in fermented milk, and their role in the modulation of the immune system that can balance the inflammatory response and increase the response to viruses (Infusino et al., [45]). Moreover, many studies have shown the importance of fruits and vegetables as rich sources of flavonoids, vitamin B, and C thus promoting antioxidant and anti-inflammatory activities, reducing risks of infection and re-infection, especially in fighting respiratory tract infections (Alkhatib, [46]; Moreb et al., [47]). Olive oil is rich in polyphenol constituents, and it is known for its anti-inflammatory property; this may provide more protection against non-communicable diseases and SARS-CoV-2 (Majumder et al., [48]). All these food groups make the pillars of a healthy and balanced diet represented by the Mediterranean diet. Di Renzo et al. [9] have shown that insufficient consumption of Mediterranean foods exposes the entire population to specific oxidative damage and, consequently, to sensitivity to COVID-19.

### Strengths and weaknesses of the study

Despite the different approaches adopted, 3% of our target population completed the questionnaire. The fact that the questionnaire was distributed only in dematerialized form may be a reason for not having a higher number of returns (this method was adopted to limit the risk of contamination). Similarly, the Beirut blast on August 4 had a significant impact on participation. The researchers decided to end the survey right after the blast in order to avoid another possible difficulty that might affect the participants’ answers.

This study has a retrospective design with inherent potential bias, particularly because it is based on two overlapping periods (economic crises and the COVID-19 pandemic). Similarly, the fact that our questionnaire appeals to the memory of the subjects could also lead to an information bias. In addition, incorrect answers may be present due to the cultural, religious, and social diversity of our sample and to difficulties in understanding the questions. In order to reduce the bias, the questionnaire was distributed in three languages and pre-tested to assess its appropriateness to the survey context. In addition, the respondents were not limited by time to complete the questionnaire, and the questions were asked in relation to very specific events. An email address was available to address participants’ concerns and questions.

## Conclusions

The results of this study showed that the pandemic and in particular the economic crisis have changed the consumption of healthy Food among hospital nurses in Lebanon. Measures are needed to improve the quality of food consumption. National authorities must work together to put in place drastic strategies to confront this alarming situation. Similar research and support may be extended to include other frontline health care workers.

## Data Availability

All of the individual participant data collected will be available after de-identification as well as other documents (Study Protocol, Statistical, Analysis Plan, Informed Consent Form, Study Report) beginning 9 months and ending 36 months following article publication. Proposals should be directed to ritag.nohra@gmail.com. To gain access, data requestors will need to sign a data access agreement.

## Abbreviations

BMI: Body mass index
PPE: Personal Protective Equipment

## Declarations

The study protocol was approved by the Ethics Committee of the Saint Joseph University of Beirut (USJ-2020-157) and the scientific committee of the Lebanese Order of Nurses and registered on clinicaltrials.gov NCT04406701.

Continued filling of the questionnaire was considered as a consent form.
